# Amyloidosis secondary to intrapulmonary Castleman disease mimicking pulmonary hyalinizing granuloma-like clinical features: A rare case report

**DOI:** 10.1097/MD.0000000000015039

**Published:** 2019-04-05

**Authors:** Shao-Ting Wang, Qi-Pu Wang, Ji Li, Ting Zhang, Lu Zhang, Yue-Ying Mao

**Affiliations:** a Department of Respiratory Medicine; b Department of Internal Medicine; c Department of Pathology; d Department of Hematology, Peking Union Medical College Hospital, Chinese Academy of Medical Science and Peking Union Medical College, Beijing, China.

**Keywords:** Castleman disease, pulmonary hyalinizing granuloma, secondary amyloidosis

## Abstract

**Rationale:**

Amyloidosis secondary to intrapulmonary Castleman disease (CD) is a rare benign disease diagnosed by histopathology. It seems to be associated with chronic inflammation, and large amounts of IL-6 produced in the germinal center of CD may enhance the production of precursor of amyloid.

**Patient concerns:**

We reported a case of an 18-year-old woman presenting with dry cough and dyspnea on exertion for 6 months and detailed exams revealed multiple pulmonary nodules, positive antinuclear antibodies, hypocomplementemia, and thrombocytopenia.

**Diagnoses:**

A computed tomography-guided percutaneous lung biopsy revealed the histopathological features of pulmonary hyalinizing granuloma (PHG), but video-assisted pulmonary wedge resection for biopsy with immunohistochemical stains finally demonstrated a corrected diagnosis of intrapulmonary CD with secondary amyloidosis.

**Interventions:**

The patient had received prednisone and Tacrolimus for 6 months.

**Outcomes:**

There was no significant improvement in pulmonary lesions or platelet level. Chemotherapy to CD was needed.

**Lessons:**

Intrapulmonary CD should be considered in patients with multiple pulmonary nodules irresponsive to corticosteroid and diagnosis of PHG should be carefully considered based on small lung biopsy sample. The treatment of amyloidosis secondary to CD remains to be uncertain.

## 1 Introduction

Castleman disease (CD), also called angiofollicular lymph node hyperplasia, is an uncommon disease of unknown etiology and intrapulmonary CD has been reported in a few cases.^[1,2]^ In 1980, Plavnick et al reported systemic amyloidosis with CD.^[3]^ The condition is fairly rare that intrapulmonary CD can mimic tumor or even metastatic adenopathy. In this case, a female patient presenting with multiple pulmonary nodules developed amyloidosis secondary to intrapulmonary CD, and was misdiagnosed as pulmonary hyalinizing granuloma (PHG) previously. In the patient, preoperative diagnosis was difficult and repeated invasive attempts, especially surgical biopsy was required for pathological diagnosis.

## 2 Case report

**Figure 1:**
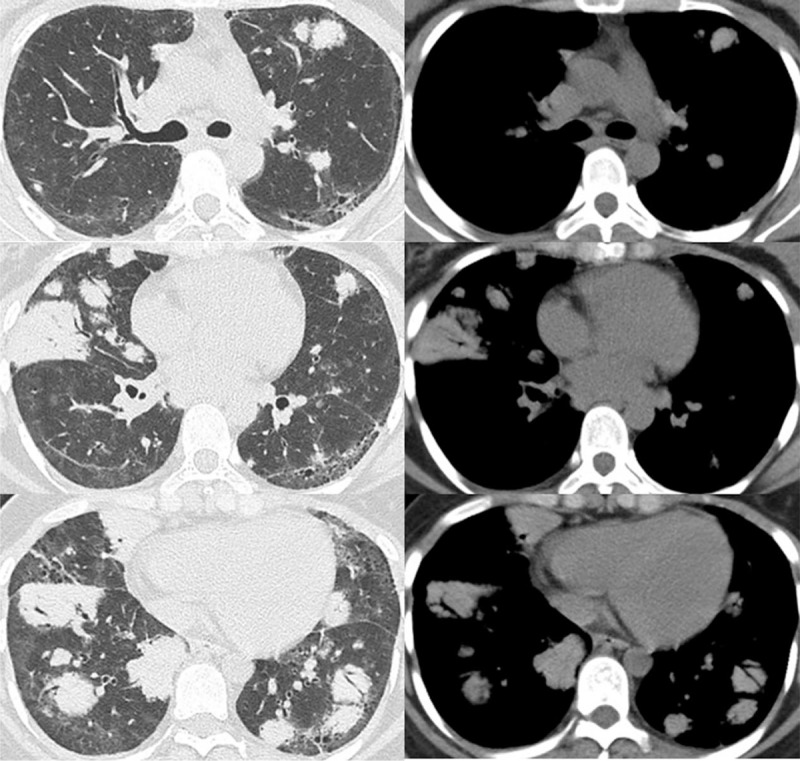
Chest CT showing multiple bilateral lung nodules. CT = computed tomography.

**Figure 2:**
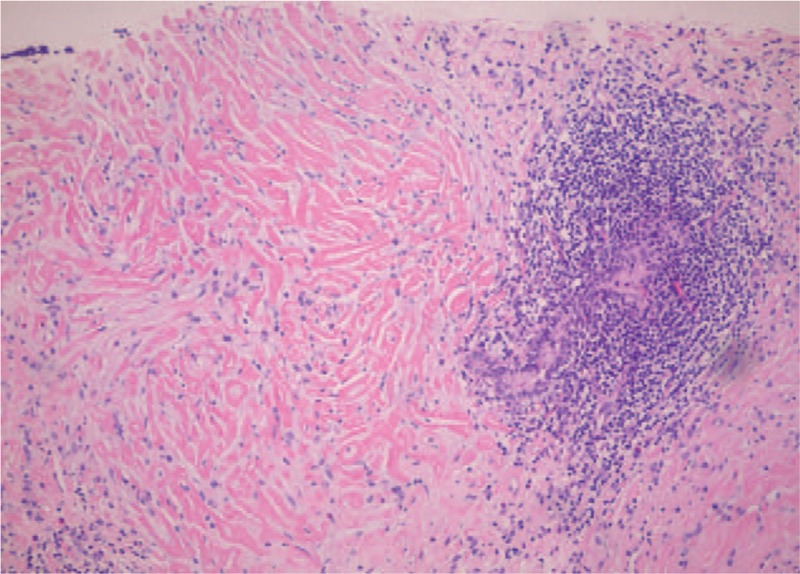
Histopathology of the pulmonary mass demonstrating bundles of hyalinized collagen fibers surrounding small vessels and lymphoplasmacytic infiltration (HE stain, ×40). HE = hematoxylin and eosin.

An 18-year-old female student was referred to our hospital presented with dry cough and dyspnea on exertion for 6 months. The patient denied fever, hemoptysis, weight loss, or tobacco use. Past medical history included immune thrombocytopenia for 3 years before the visit, for which she received 1.5 years of oral glucocorticoid. Laboratory examinations revealed iron deficiency anemia (hemoglobin = 90 g/L), platelet count of 6 × 10^9^/L, and total white cell count of 4.4 × 10^9^/L. Urinalysis, liver function, electrolytes, and creatinine were within normal ranges. Her erythrocyte sedimentation rate (>140 mm/h), C-reactive protein (150.1 mg/L), and serum IL-6 (7.3 pg/mL), IL-8 (279 pg/mL), tumor necrosis factor-α (175.0 pg/mL) were significantly elevated. Serum measurement of immunoglobulins showed elevated total IgG of 27.59 g/L, while serum IgG4, M protein, serum, and urine immunofixation electrophoresis and light chain were negative or normal. Hypocomplementemia (C4 = 0.079 g/L), positive antinuclear antibodies (titer 1:160), and positive Coombs test were found. Additional clinically relevant autoantibodies, (1–3)-β-D-glucan, galactomannan, interferon gamma release assay, and tumor markers were negative or normal. Pulmonary function tests revealed restriction and decreased diffusing capacity with forced vital capacity of 2.05 L (65.9% predicted), total lung capacity of 2.83 L (68.9% predicted) and diffusing capacity for carbon monoxide of 28.8% predicted. Chest computed tomography (CT) showed multiple well-defined nodules randomly distributed in both lung fields and mediastinum lymphadenopathy, with no significant pleural effusion (Fig. 1). Bronchoscopy demonstrated no endobronchial pathological lesions, and a culture of bronchoalveolar lavage fluid showed no evidence of tuberculosis or fungal infection. Cytological evaluation was negative for malignant cells. A CT-guided percutaneous lung biopsy was performed, and histopathological examinations revealed some defining features for hyalinizing granuloma, including homogenous hyaline lamellae around small blood vessels and dense infiltrates of lymphocytes, plasma cells with frequent lymph follicle formation (Fig. 2). The patient was diagnosed with PHG, and received prednisone 1 mg/kg/d and tacrolimus. Unfortunately, 6 months after initiation of therapy with prednisone gradually tapered, neither pulmonary lesions nor platelet level revealed remarkable interval change. As detailed investigations in clinic failed to uncover any more underlying condition, the patient underwent video-assisted pulmonary wedge biopsy.

**Figure 3:**
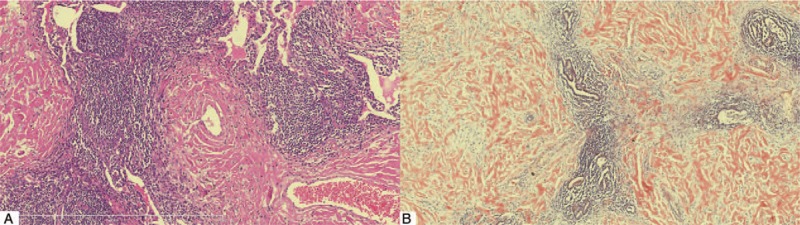
Histopathology of video-assisted pulmonary wedge resection. (a) Histopathology exam showing nodular interstitial pink deposits surrounding small vessel, lymphoid tissue hyperplasia, and follicular architecture along bronchovascular bundle, with abundant plasma cells scattered (HE stain, ×200). (b) Light microscopy demonstrating alcoholized Congo red positive amyloid deposits within pulmonary interstitium (alcoholized Congo red strain, ×200). HE = hematoxylin and eosin.

Intraoperatively, multiple white and tough-elastic nodules were found in the upper and lower lobes of lung, partially fused to mass, with pulmonary parenchyma edema surrounded. One isolated lesion was dissected from the left upper lobe, which contained 2 nodules. The diameters of nodules were 1 and 2 cm, and specimens were sent for microorganism culture and pathological examination, respectively. Microorganism culture was negative. Pathologic exam revealed nodular interstitial pink deposits in lungs which stained pink with hematoxylin and eosin, and pathologic examination also showed lymph node hyperplasia and follicular architecture along broncho vascular bundle and under the pleura, with plasma cell infiltration (Fig. 3a). On immunohistochemical stains, the hyperplastic cells were positive for CD20 (follicle), CD138 and negative for CD3, IgG, and IgG_4_. Congo-red, alcoholized Congo-red (Fig. 3b), alkaline Congo-red stains, and kappa and lambda light chain immunostains were positive. Histological evaluation demonstrated a revised diagnosis of intrapulmonary CD with secondary amyloidosis. Human herpesvirus-8 (HHV-8) was tested to be negative.

## 3 Discussion

CD has been described as a lymphoproliferative disorder that features benign hyperplasia of the lymph nodes, and the etiology is not known with certainty. The plasma cell type and the mixed type are accompanied with various systemic manifestations, such as fever, weight loss, anemia, splenomegaly, peripheral lymphadenopathy, hypoalbuminemia, hypergammaglobulinemia, and increased levels of acute-phase proteins. In 2017, an international panel established the first ever diagnostic criteria for idiopathic multicentric Castleman disease (iMCD), which was HHV-8-negative, and the criteria required multicentric lymphadenopathy with defined histopathology, ≥2 clinical/laboratory changes, and exclusion of iMCD mimics.^[4]^ The recently described “TAFRO syndrome” identifies a subset of iMCD patients with shared manifestations, including thrombocytopenia, anasarca/ascites, reticulin fibrosis in bone marrow, renal dysfunction, organomegaly (TAFRO), while iMCD patients without TAFRO syndrome typically have thrombocytosis, hypergammaglobulinemia, and less severe fluid accumulation.^[5]^

The relationship between CD and secondary amyloidosis was reported before, and most cases revealed amyloid deposits in kidney.^[6–9]^ Secondary amyloidosis is believed to be caused mainly by chronic inflammation, and large amounts of IL-6 produced in the germinal center of CD may enhance the production of precursor of amyloid.^[10,11]^

Intrapulmonary CD is exceedingly rare,^[12]^ and differential diagnosis includes all types of tumors, like lung carcinoma, carcinoid tumors, hamartochondroma, lymphoma, or metastases. While our patient was misdiagnosed as PHG previously, PHG is a rare benign disease and respiratory manifestations were reported such as cough, chest pain, dyspnea, and hemoptysis. Extrapulmonary fibrous lesions, including skin, pharynx, pleural, pericardium, liver, may accompany PHG. About 70% of patients present with multiple lung nodules, sometimes with calcification and cavity, and F-18-fluorodeoxyglucose positron emission tomography can reveal hypermetabolic activity in PHG lesions.^[13–15]^ PHG seems to be associated with autoimmune diseases with some serologic evidences,^[16,17]^ and infection and lymphoproliferative disorders are also assumed to be the cause.^[18]^ Corticosteroids and immunosuppressive drug were effective in some cases.^[19]^ Whether PHG represents a specific pathologic diagnosis or a clinicopathologic entity remains to be explored. PHG is diagnosed based on histopathology, which is characterized by a central area of whorled hyalinized collagen lamellae surrounded by lymphocytes, plasma cells, and histiocytes,^[20]^ but the diagnosis of PHG based on the needle biopsy might be questionable. Our patient, that the diagnosis of CD and secondary amyloidosis was unclear until the histopathological examination of the resected lymphoid tissue, and fulfilled the diagnostic criteria of iMCD. In agreement with previous reports, bronchoscopy or a pulmonary fine needle biopsy might not provide a definitive diagnosis of CD, and surgical resection and immunostains were finally needed.

The therapeutic effect of corticosteroid with tacrolimus was poor for our patient. Untreated systemic amyloidosis often has an unrelenting clinical course, and the treatment of amyloidosis secondary to CD is presently unknown. There has been a steady increase in survival with secondary reactive amyloidosis in treatment strategies for underlying inflammatory disorders.^[21]^ Complete surgical resection was proposed to be the best treatment for unicentric CD. However, the role of surgery in multicentric disease is limited, and the efficacy of systemic therapy, such as radiotherapy, steroids alone, or together with antineoplastic chemotherapy, even humanized anti-IL-6 receptor monoantibody, is uncertain.^[22]^

This case illustrates the clinical features of intrapulmonary CD with secondary amyloidosis mimicking PHG. The limitations in approach to this study is etiology, physiopathology, and treatment of amyloidosis secondary to CD remain to be further investigated and the patient needed longer follow-up.

## Author contributions

STW and QPW designed the case report and were major contributors in writing the manuscript, they contributed equally to this work. JL acquired and interpreted the patient's pathological data. TZ collected the patient clinical data. LZ re-evaluate the diagnosis of CD and YYM checked the bone marrow smear of patient. JHS reviewed and revised the manuscript.

**Data curation:** Qi-Pu Wang, Ting Zhang, Lu Zhang, Yue-Ying Mao.

**Investigation:** Shao-Ting Wang, Ji Li.

**Resources:** Yue-Ying Mao.

**Writing – original draft:** Qi-Pu Wang.

**Writing – review and editing:** Shao-Ting Wang, Ting Zhang, Lu Zhang.
